# The Effects of Time-Restricted Eating in Women with Polyendocrine Metabolic Ovarian Syndrome: A Systematic Review and Meta-Analysis

**DOI:** 10.3390/nu18132096

**Published:** 2026-06-26

**Authors:** Mohammed Hamsho, Meriem Bensaoua, Wijdan Shkorfu, Yazan Ranneh, Faiza Kalam

**Affiliations:** 1 Department of Nutrition and Dietetics, Faculty of Health Science, Istanbul Yeni Yuzyil University, Istanbul 34010, Turkey; mohammed.hamsho@hotmail.com; 2 Department of Nutrition and Dietetics, Faculty of Health Science, Istanbul Istinye University, Istanbul 34396, Turkey; meriembensaoua@gmail.com; 3 Independent Researcher, Istanbul 34400, Turkey; wijdanshkorfu@gmail.com; 4 Department of Nutrition and Dietetics, College of Pharmacy, Al-Ain University, Abu Dhabi P.O. Box 64141, United Arab Emirates; yazan.ranneh@aau.ac.ae; 5 Division of Cancer Prevention and Control, Department of Internal Medicine, College of Medicine, The Ohio State University Comprehensive Cancer Center, Columbus, OH 43214, USA; 6 Division of Cancer Prevention and Control, Department of Internal Medicine, The Ohio State University, Columbus, OH 43214, USA

**Keywords:** polycystic ovary syndrome, polyendocrine metabolic ovarian syndrome, PMOS, time-restricted eating, intermittent fasting, systematic review, meta-analysis, insulin resistance, SHBG, circadian rhythm

## Abstract

**Background:** Polyendocrine metabolic ovarian syndrome (PMOS), formerly known as polycystic ovary syndrome (PCOS), is a complex endocrine–metabolic disorder strongly associated with insulin resistance, hyperandrogenism, and obesity. Time-restricted eating (TRE) has emerged as a promising dietary strategy for improving metabolic health; however, evidence regarding its efficacy in women with PMOS remains limited. **Objective:** To systematically evaluate the effects of TRE on metabolic, hormonal, anthropometric, and adherence-related outcomes in women with PMOS. **Methods:** PubMed, Embase, Scopus, and Web of Science were systematically searched through 25 April 2026, for randomized controlled trials evaluating TRE interventions in women with PMOS. Random-effects meta-analyses were performed to pool mean differences (MD) with 95% confidence intervals for specific metabolic, hormonal, and anthropometric outcomes. **Results:** Four randomized controlled trials comprising 216 women with PMOS were included; three trials compared TRE with calorie restriction and one with ad libitum intake. Compared with control interventions, TRE significantly improved HOMA-IR (MD = −0.58, 95% CI: −0.87 to −0.30), QUICKI (MD = 0.08, 95% CI: 0.04 to 0.13), and HDL (MD = 1.97 mg/dL, 95% CI: 0.96 to 2.99). TRE was generally associated with high adherence across the included studies, with some trials reporting higher compliance than calorie restriction. **Conclusions:** Current evidence from four RCTs suggests that TRE may serve as a promising alternative dietary strategy for women with PMOS, particularly for improving insulin sensitivity. However, the evidence remains limited by the small number of available studies, modest sample sizes, and heterogeneity across interventions. Therefore, these findings should be considered preliminary and require confirmation in a larger, longer-term randomized controlled trial.

## 1. Introduction

Polycystic ovary syndrome (PCOS) is a common, complex metabolic disorder characterized by hyperandrogenism, ovulatory dysfunction, and frequent insulin resistance (IR). These endocrine and metabolic abnormalities frequently manifest as acne, hirsutism, menstrual irregularities, and adverse metabolic features, substantially affecting quality of life [1,2,3]. PCOS also confers an increased risk of type 2 diabetes, and metabolic dysfunction-associated steatotic liver disease [4,5].

Recently, an international consensus process proposed the term “Polyendocrine Metabolic Ovarian Syndrome” (PMOS) as an alternative nomenclature intended to better reflect the complex endocrine and metabolic features of the disorder. However, PCOS remains the most widely recognized and commonly used term in current clinical practice, guidelines, and the current scientific literature. For consistency with the recent consensus publication, the term PMOS is used throughout the present manuscript [6].

PMOS is a multifactorial endocrine–metabolic disorder that cannot be attributed to a single etiological factor. Several interacting contributors, including poor dietary patterns, excessive caloric intake, increased adiposity, gut dysbiosis, and genetic predisposition, are thought to underlie its development [2,7]. At the molecular level, IR—largely driven by these factors—stimulates ovarian theca cells to increase androgen production, ultimately contributing to the characteristic clinical manifestations of PMOS [8]. Although pharmacological therapies such as metformin are commonly used in PMOS management, lifestyle modification remains the cornerstone of treatment, highlighting the need to optimize dietary and behavioral interventions [9].

Dietary intervention plays a central role in PMOS management, particularly in women with overweight or obesity, in whom modest weight reduction can improve insulin sensitivity, hyperandrogenism, ovulatory function, and broader metabolic outcomes. In this context, Intermittent fasting (IF), an eating pattern characterized by alternating periods of fasting and feeding, has gained considerable popularity due to its association with a range of short- and long-term health benefits. During fasting periods, energy intake is restricted or completely avoided, whereas caloric consumption is confined to designated feeding windows. Several IF regimens have been investigated, with the most commonly studied being time-restricted eating (TRE), alternate-day fasting (ADF), and the 5:2 fasting approach [10,11]. Systematic reviews suggest that IF interventions may improve anthropometric and metabolic parameters in women with PMOS, although substantial heterogeneity exists across fasting protocols and study designs [12,13,14].

Unlike ADF and 5:2 approaches, TRE—characterized by limiting daily food intake to specific eating windows without necessarily prescribing continuous caloric restriction—has gained particular interest in the scientific field due to its ability to improve metabolic health [15,16]. Current evidence suggests that TRE may improve circadian alignment, glucose metabolism, and insulin sensitivity, which are closely implicated in PMOS pathophysiology. Women with PMOS may therefore represent a particularly relevant target population for TRE interventions, as several proposed mechanisms of TRE directly address key metabolic abnormalities underlying the disorder, including insulin resistance, obesity, and circadian dysregulation. Therefore, the present meta-analysis aimed to systematically evaluate the effects of TRE interventions on anthropometric, metabolic, and hormonal outcomes, as well as adherence, in women with PMOS.

## 2. Methods

We conducted this systematic review in accordance with the Preferred Reporting Items for Systematic Reviews and Meta-Analyses (PRISMA) [17]. The study protocol was registered in PROSPERO (CRD420261389630), on 17 May 2026.

### 2.1. Search Strategy

We systematically searched PubMed, Web of Science, Embase, and Scopus for inception through 25 April 2026, to identify randomized controlled trials (RCTs) evaluating the effect of TRE in women with PMOS. Search terms combined PMOS-related terms and TRE-related keywords, and the complete search strategy is provided in Table S1. In addition, we manually screened the reference lists of eligible articles to identify any further relevant studies.

### 2.2. Eligibility Criteria

Studies were included if they met the following criteria: (1) **Population**: women diagnosed with PMOS according to recognized criteria (e.g., Rotterdam, NIH, or AE-PCOS); (2) **Intervention**: TRE, defined as a daily eating window of ≤10 h, with or without calorie restriction, for a minimum duration of 2 weeks; (3) **Comparison**: ad libitum or calorie-restricted diets, with the calorie-restricted group preferentially selected when multiple comparator arms were available, as CR represents the current first-line dietary intervention for PMOS and provides a more clinically relevant comparator for evaluating the effects of TRE; (4) **Outcome**: evaluation of at least one prespecified metabolic, anthropometric, or adherence-related outcome; (5) **Study design:** RCT, peer-reviewed, published as full-text articles in the English.

We excluded studies conducted in animal or in vitro models, as well as case reports, narrative reviews, systematic reviews, meta-analyses, conference abstracts, letters, or editorials.

### 2.3. Outcomes

The primary outcomes of this meta-analysis were changes in:

- Glucoregulatory markers: fasting blood glucose (FBG), fasting blood insulin (FBI), hemoglobin A1C (HbA1c), and Homeostatic Model Assessment for Insulin Resistance (HOMA-IR), and the Quantitative Insulin Sensitivity Check Index (QUICKI). Within the glucoregulatory domain, HOMA-IR and QUICKI were considered the key indices of insulin sensitivity. Other glycemic markers (FBG, FBI, HbA1c) and the remaining lipid and hormonal measures were prespecified as secondary or exploratory outcomes, rather than co-primary endpoints.
- Lipid profile such as triglycerides (TGs), low-density lipoprotein (LDL) cholesterol, high-density lipoprotein (HDL) cholesterol, and total cholesterol (TC).
- Hormonal markers: such as total testosterone (TT), free androgen index (FAI), sex hormone-binding globulin (SHBG), luteinising hormone (LH), and follicle-stimulating hormone (FSH). Secondary outcomes included body weight, body mass index (BMI), waist circumference (WC), body fat, hirsutism, acne, menstrual outcomes, and intervention adherence.

### 2.4. Study Selection

Two reviewers (W.S and M.B.) independently and in duplicate screened all titles and abstracts against the predefined eligibility criteria. In the second stage, full texts of potentially eligible articles were assessed in detail. Any discrepancies between reviewers were resolved through discussion or, when necessary, by consultation with a third investigator (M.H.). The study selection process was documented using a PRISMA flow diagram, and reasons for exclusion were recorded at each stage.

### 2.5. Data Extraction

Data extracted from each included study comprised study identifier, country, sample size, study design, study duration, mean age, baseline BMI, TRE intervention details, and comparator diet details. Extraction was performed independently by two authors (W.S. and M.B.), and discrepancies were resolved by a third author (M.H.). For the assessment of intervention effects, prespecified outcome data were extracted for each intervention and comparator arm—including mean changes from baseline and corresponding standard deviation (SD)—or these values were calculated using appropriate statistical methods when not directly reported. Study screening and data extraction were managed using Covidence systematic review software (Veritas Health Innovation, Melbourne, Australia).

### 2.6. Quality Assessment

The methodological quality of included RCTs was assessed using the Cochrane Risk of Bias 2 (RoB2) tool [18]. Risk of bias was evaluated at the outcome level across five domains: (1) bias arising from the randomization process, (2) bias due to deviations from intended interventions, (3) bias due to missing outcome data, (4) bias in measurement of outcome, and (5) bias in selection of the reported results. Each domain was rated as “low risk”, “some concerns”, or “high risk” of bias according to RoB 2 guidance, and overall risk of bias was determined by the highest level of risk identified across the domains. Two reviewers (Y.R. and M.B.) independently performed the assessments, with disagreements resolved through discussion or, when necessary, adjudication by a third author (M.H.).

### 2.7. Statistical Analysis

The present meta-analysis was performed using Review Manager (RevMan) Version 5.4 (Cochrane Collaboration). Mean and standard deviation (SD) values of change from baseline for prespecified outcomes were extracted from the included studies. Mean difference (MD) was used when outcomes were reported on the same scale; otherwise, standardized mean difference (SMD) was applied.

When change from baseline mean and SDs were not directly reported, they were derived using appropriate methods. If the median with first and third quartile (Q1, Q3) were provided, values were converted to the mean and SDs according to the method described by Wan et al. [19]. When standard error of the mean (SEM) was reported, SD was calculated as: SD = SEM × √n, where n is the number of participants. When the mean was presented with 95% confidence intervals, SD was estimated as: SD = √n × (upper limit − lower limit)/3.92. Due to insufficient reporting of paired-data statistics, the crossover study was analyzed as a parallel-group comparison using the available data [20].

Pooled analyses were presented as MD or SMD with 95% confidence intervals (CI). Where necessary, outcome units were converted to a common scale prior to pooling to ensure comparability across studies and allow the use of MDs in the meta-analysis. Pooled estimates were generated using fixed-effect models when heterogeneity was low (I^2^ < 50%) and random-effects models when heterogeneity was moderate to high (I^2^ ≥ 50%. Interstudy heterogeneity was assessed using Cochran’s Q and the I^2^ statistics; I^2^ values of ≤50% and ≥75% were interpreted as indicating moderate and considerable heterogeneity, respectively [21,22]. Statistical significance was defined as two-sided *p* < 0.05. Because only four randomized trials were eligible and sample sizes were small, we were not able to perform meaningful subgroup or meta-regression analyses to formally investigate potential sources of heterogeneity. A sensitivity analysis was performed by excluding the only study that employed an ad libitum comparator and crossover design to evaluate the robustness of the pooled estimates.

## 3. Results

### 3.1. Literature Selection

**Figure 1:**
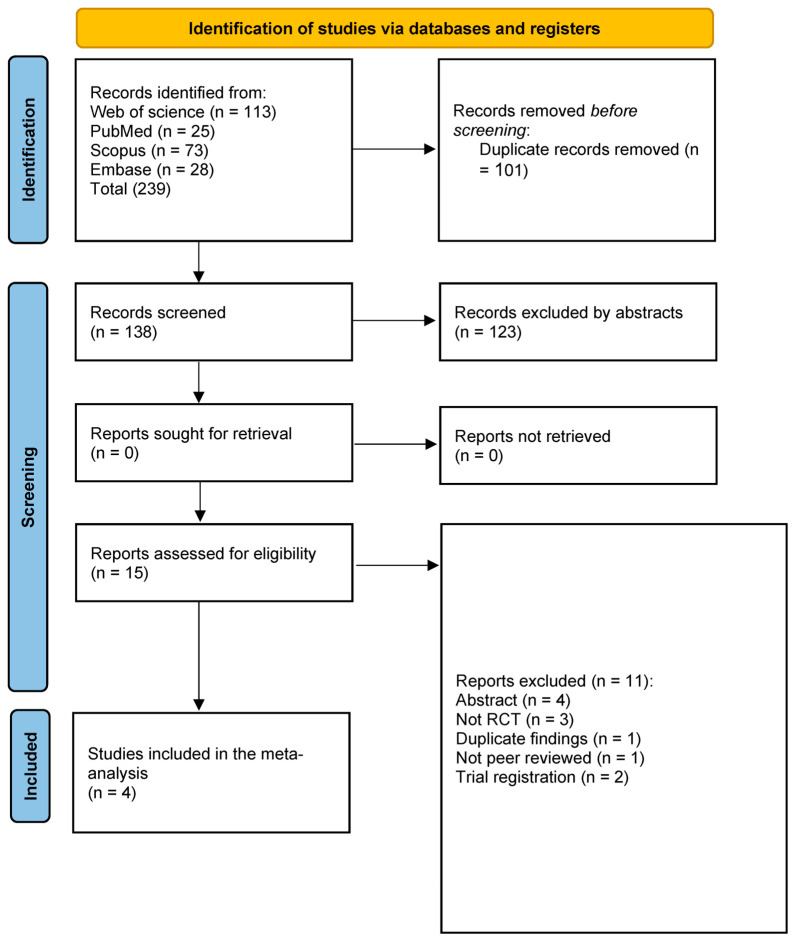
Prisma Flow Chart.

The initial search identified 239 records. After removal of 101 duplicates, 138 unique records remained. Following title and abstract screening, 123 records were excluded based on the prespecified eligibility criteria. The full text of 15 articles was assessed, of which 11 were excluded for not meeting the inclusion criteria. Supplementary Table S2 provides the title of excluded studies and reasons for exclusion. Thus, 4 RCTs were included in this meta-analysis (Figure 1).

### 3.2. Studies’ Characteristics

Key characteristics of the included trials are summarized in Table 1. The studies were conducted in a diverse geographic setting, including China, Ireland, Iran, and the United States. All four trials enrolled women diagnosed with PMOS according to the Rotterdam Criteria, requiring at least two of the following: oligo-anovulation, clinical or biochemical hyperandrogenism, and polycystic ovarian morphology. Trials durations ranged from 2 to 6 months. TRE interventions employed eating windows from 6, 8, or 10 h. Two trials [23,24] evaluated early TRE protocols starting between 08:00 and 09:00, whereas Corapi et al. implemented a later window starting at 13:00 [25]. It should be noted that while Corapi et al. included three arms (TRE, CR, and ad libitum), only TRE and CR arms were included in the meta-analysis. Accordingly, three trials contributed comparisons of TRE versus CR, and one trial compared TRE with ad libitum intake [26].

### 3.3. Risk of Bias Assessment

Overall, three studies were judged as having “some concerns” regarding risk of bias, primarily due to insufficient reporting of allocation concealment, incomplete outcome data, and limited information on the handling of missing data. The remaining trial was considered at low risk of bias across all RoB 2 domains (Supplementary Figure S1).

### 3.4. Glucose Metabolism

**Figure 2:**
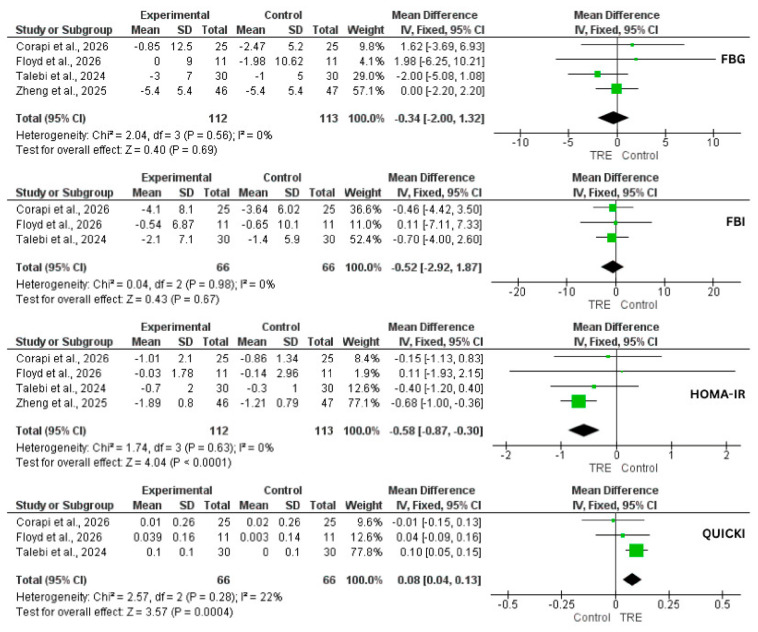
Forest plots for the FBG (mg/dL), fasting blood insulin (mU/L), HOMA-IR, and QUICKI of time-restricted eating (TRE) vs. control.

The TRE and control groups showed comparable changes in FBG (MD = −0.34 mg/dL, 95% CI = −2.00, 1.32, *p* = 0.69, I^2^ = 0%) and FBI (MD = −0.52 mU/L, 95% CI = −2.92, 1.87, *p* = 0.67, I^2^ = 0%). In contrast, HOMA-IR was significantly reduced in the TRE group compared with controls (MD = −0.58, 95% CI = −0.87, −0.30, *p* < 0.0001, I^2^ = 0%). QUICKI values were also significantly higher following TRE (MD = 0.08, 95% CI = 0.04, 0.13, *p* = 0.004, I^2^ = 22%) (Figure 2). These findings were further supported by HbA1c outcomes, which was assessed in two studies, both of which reported significant reductions in the TRE group compared with the control group [25,26].

### 3.5. Lipid Profile

**Figure 3:**
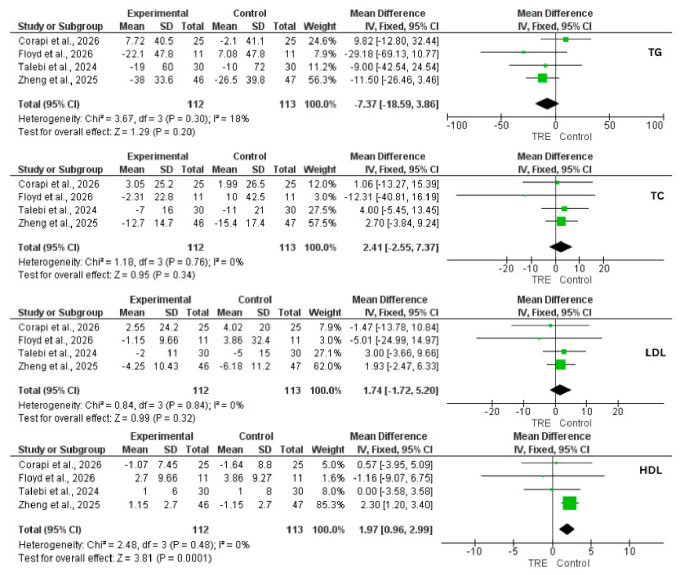
Forest plots for the TG (mg/dL), TC (mg/dL), LDL (mg/dL), and HDL (mg/dL) of time-restricted eating (TRE) vs. control.

No significant differences were observed between the TRE and control groups for TG (MD = −7.37 mg/dL, 95% CI = −18.59, 3.86, *p* = 0.20, I^2^ = 18%), TC (MD = 2.41 mg/dL, 95% CI = −2.55, 7.37, *p* = 0.34, I^2^ = 0%), or LDL cholesterol (MD = 1.74 mg/dL, 95% CI = −1.72, 5.20, *p* = 0.32, I^2^ = 0%). In contrast, HDL cholesterol levels were significantly higher in the TRE group (MD = 1.97 mg/dL, 95% CI = 0.96, 2.99, *p* = 0.0001, I^2^ = 0%) (Figure 3). Floyd et al. additionally evaluated apolipoprotein A and B concentrations; however, there were no significant between-group differences detected [26].

### 3.6. Sex Hormones

**Figure 4:**
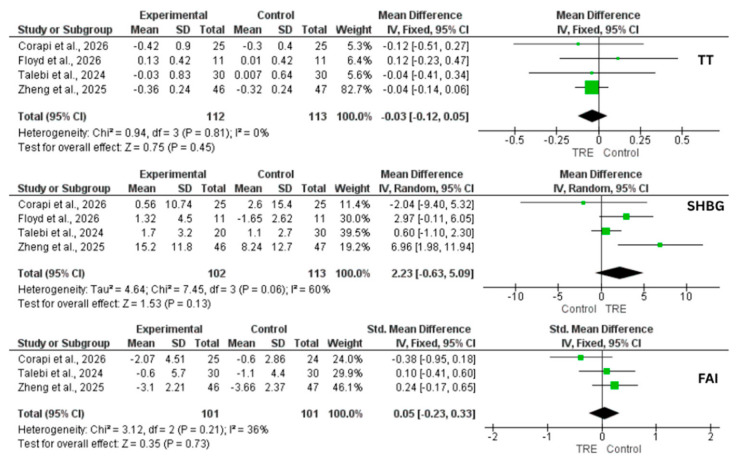
Forest plots for the TT (nmol/L), SHBG (nmol/L), and FAI of time-restricted eating (TRE) vs. control.

There were no significant differences between the TRE and control groups in TT (MD = −0.03 nmol/L, 95% CI = −0.12, 0.05, *p* = 0.45, I^2^ = 0%) or FAI (SMD = 0.05, 95% CI = −0.23, 0.33, *p* = 0.73, I^2^ = 36%). Similarly, SHBG concentrations did not differ between the groups (MD = 2.23 nmol/L, 95% CI = −0.63, 5.09, *p* = 0.13, I^2^ = 60%) (Figure 4). We additionally assessed dehydroepiandrosterone sulfate, androstenedione, free testosterone, and 17-hydroxyprogesterone, with no significant between-group differences observed [26].

### 3.7. Anthropometric Measures

**Figure 5:**
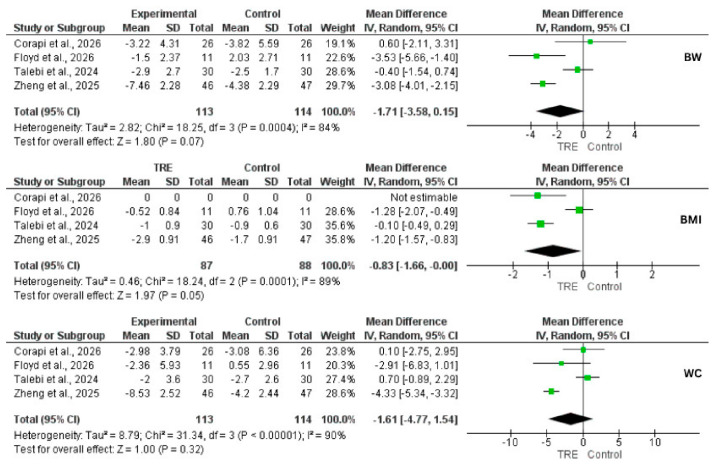
Forest plots for the body weight (kg), body mass index (kg/m^2^), and waist circumference (cm) of time-restricted eating (TRE) vs. control.

Body weight (BW) and waist circumference (WC) were reported in all four trials, including 113 women in the TRE group and 114 in the control group. No significant between-group differences were observed, although substantial heterogeneity was present (BW: MD = −1.71, 95% CI = −3.58, 0.15, *p* = 0.07, I^2^ = 84%; WC: MD = −1.61 cm, 95% CI = −4.77, 1.54, *p* = 0.32, I^2^ = 90%). A similar pattern was observed for BMI (MD = −0.83 kg/m^2^, 95% CI = −1.66, −0.00, *p* = 0.05, I^2^ = 89%) (Figure 5). Zheng et al. reported a greater reduction in fat mass with TRE compared with usual care (−5.5 vs. −3.8 kg), although the between-group difference was not statistically significant (*p* = 0.130) [23]. Similarly, Corapi et al. observed comparable reductions in fat mass between the TRE and calorie restriction groups over 6 months (−3.71 vs. −4.16 kg; *p* = 0.69) [25].

### 3.8. Clinical Manifestations of PMOS

Hirsutism was evaluated in three trials including 102 women in the TRE group and 103 in the control group, with no significant between-group differences observed (MD = −0.18, 95% CI = −0.46, 0.09, *p* = 0.20, I^2^ = 0%) (Supplementary Figure S2). Acne outcomes were assessed in two trials [25,26], neither of which demonstrated significant changes compared with the baseline or control groups. Conversely, Talebi et al. reported significant improvements in acne severity in both the TRE and CR groups relative to baseline, although no significant difference was detected between interventions [24]. These changes may reflect the high baseline acne scores in that study. Menstrual cycle interval and menstrual blood loss were evaluated by Corapi et al., with no significant changes observed [25].

### 3.9. Adherence, Fasting-Feasting Windows, and Total Caloric Intake

Adherence measurement and reported compliance rates varied across the trials, but TRE generally showed high feasibility. In the 16-week trial by Zheng et al., adherence was defined as the average number of adherent days per month, and participants reported 21.8 ± 6.1 days/month in the TRE group and 22.8 ± 6.9 days/month in the control group, with no meaningful changes in caloric intake or macronutrient distribution between the groups [23].

Floyd et al. defined compliance as the proportion of days that participants strictly maintained a 6 h window; completers achieved a mean compliance rate of 94.7% [26]. In the 8-week trial by Talebi et al., non-compliance was defined as failing to follow instructions three times/week for more than two consecutive weeks. The TRE groups ate *ad libitum* within their window, whereas the CR control group was prescribed a 25% energy reduction (averaging 1500–1800 kcal/day). By the end of the study, the TRE groups achieved spontaneous energy restriction of about 300 kcal/day, with no significant differences in macronutrient composition compared to the CR group [24].

Corapi et al. defined adherence for TRE as eating within 1 h of the prescribed 6 h window and for CR as remaining within 200 kcal of the daily goal. The TRE group was adherent on 80% of days (5.6 days per week), notably higher than the 60% adherence in the CR group. Both groups achieved an approximate 10% caloric deficit relative to baseline. In the TRE group, the actual eating window decreased from 9:25 ± 1:38 at baseline to 5:55 ± 1:56 by the end of the 6-month trial, whereas the CR group maintained a similar eating window (9:48  ±  1:50) with no substantial change from baseline [25].

### 3.10. Sensitivity Analysis

A sensitivity analysis excluding the only study with an ad libitum comparator and crossover design yielded results that were largely consistent with the primary analysis, indicating that the overall findings were not materially influenced by the inclusion of this study.

## 4. Discussion

To the best of our knowledge, this is the first systematic review and meta-analysis assessing the effectiveness of TRE in women with PMOS. Our findings suggest that TRE may improve selected metabolic and endocrine abnormalities associated with PMOS, particularly markers of insulin sensitivity, but the robustness and certainty of these effects are limited by the small number of available RCTs, modest overall sample size, and short follow-up. Although caloric intake was matched across most studies, the findings raise the possibility that TRE may confer benefits beyond those attributable to caloric restriction and weight loss alone; however, this hypothesis requires confirmation in larger trials.

Despite the absence of significant between-group differences in body weight in the pooled analysis, individual trials demonstrated considerable heterogeneity. Talebi et al. and Corapi et al. reported comparable weight loss between TRE and daily CR groups, noting that the caloric intake in TRE groups was reduced spontaneously due to the constrained eating window rather than explicit calorie counting [24,25]. In contrast, Zheng et al. observed a significantly greater weight loss in the TRE group (7.47 kg vs. 4.38 kg), despite both arms reporting nearly identical daily energy intake during the intervention. This discrepancy may reflect the limitations of self-reported dietary intake, including underreporting of energy consumption [23,26]. These findings differ from larger meta-analyses in broader populations, which have reported greater weight loss with TRE compared with CR [27,28].

Although the pooled analysis did not demonstrate a significant effect of TRE on SHBG concentrations, SHBG remains a clinically relevant marker because of its association with both insulin sensitivity and androgen bioavailability [26]. While weight loss alone is constantly associated with higher SHBG levels, emerging evidence suggests that these hormonal changes may also be influenced by the timing of energy intake, potentially via improved circadian alignment, independent of weight reduction. These mechanisms may be additive when combined. Several findings support this dual contribution of weight loss and meal timing. In a post hoc analysis by Corapi et al., participants who achieved at least 5% weight loss experienced greater improvements in SHBG and menstrual regularity, irrespective of whether they followed TRE or CR [25]. Conversely, Li et al. reported that instructing women with PMOS to consume all meals between 8:00 and 16:00 led to significant increases in SHBG despite only ~1% body weight reduction [29]. Similarly, Jakubowicz et al. found that in lean women with PMOS, concentrating energy intake earlier in the day (at breakfast rather than dinner) produced a marked (~105%) increase in SHBG [30].

The potential synergy between weight loss and circadian alignment is further illustrated by Zheng et al., in which the TRE group experienced a substantially greater increase in SHBG (+15.21 nmol/L) compared with the CR group (+8.25 nmol/L), alongside more pronounced weight loss. The magnitude of SHBG improvement observed in this trial exceeded that reported in several other included studies [23]. Complementary evidence from a case report of monozygotic twin in who followed a 16:8 TRE regimen (8:00–16:00) combined with CR produced weight loss and an increase in SHBG comparable to those observed by the Zheng et al. study [31]. In contrast, a RCT of two TRE schedules (15:00–19:00) vs. 13:00–19:00) in pre and postmenopausal women found no effect on SHBG levels after 8 weeks of intervention despite 3–4% weight loss [32].

Notably, these findings also highlight the potential importance of distinguishing between early and late TRE protocols. Studies reporting favorable changes in SHBG implemented eating windows aligned with the earlier part of the day (e.g., 8:00–16:00), whereas the trial evaluating later eating windows (13:00–19:00 and 15:00–19:00) found no significant effect on SHBG. Recent systematic reviews and network meta-analyses have reported greater improvements in insulin sensitivity with eTRE compared with lTRE across broader populations, particularly among individuals with dysglycemia (e.g., insulin resistant and T2D) [33,34]. Although direct comparisons between eTRE and lTRE in women with PMOS are currently lacking, the well-established association between insulin resistance and reduced SHBG production suggests that the timing of the eating window may be an important determinant of endocrine and metabolic outcomes in this population. Future studies should directly compare eTRE and lTRE protocols to clarify their relative effects on insulin sensitivity and SHBG concentrations. Collectively, these observations support the hypothesis that both weight loss and meal timing may influence SHBG concentrations; however, the available evidence is sparse and indirect, and the overall certainty regarding these mechanisms in PMOS remains low. The bidirectional relationship between IR and PMOS is well established and is further exacerbated by obesity and excess visceral adiposity. In addition, chronic low-grade inflammation associated with excess adiposity may further impair insulin signaling and contribute to the metabolic dysfunction characteristic of PMOS. This metabolic milieu stimulates ovarian androgen production while suppressing hepatic SHBG synthesis [8,35]. In our analysis, FBG and FBI did not differ significantly between groups, but HOMA-IR improved significantly with the TRE, accompanied by increases in QUICKI, indicating enhanced insulin sensitivity. Although the mechanisms underlying these improvements remain incompletely understood, modulation of metabolic and inflammatory pathways has been proposed as a potential contributor to the beneficial effects of dietary interventions. Despite this, the clinical implications of changes of this magnitude in women with PMOS remain uncertain.

An important observation is that TRE appears to influence overall glycemic control rather than only fasting values. This is supported by reductions in HbA1c in TRE groups in the included trials and by studies using continuous glucose monitoring in individuals with type 2 diabetes, where TRE has been associated with lower 24 h glucose exposure [36,37]. These findings are particularly relevant for women with PMOS, who frequently exhibit dysglycaemia and have an elevated risk of progression to type 2 diabetes [4].

In contrast, we observed no significant between-group differences in most lipid parameters, supporting the notion that lipid changes are primarily driven by the magnitude of weight loss rather than the timing of food intake. HDL cholesterol was an exception, showing significant increases in the pooled analysis, a phenomenon that aligns with metabolic responses to TRE observed in other populations [38]. However, this effect was largely driven by the Zheng et al. study, which carried 85% of the overall effect [23]. Accordingly, the HDL findings should be interpreted cautiously until replicated in larger, independent trials. Given the very small number of available studies, formal leave-one-out sensitivity analyses for HDL-cholesterol would reduce the pooled estimate to a small subset of underpowered trials and were therefore not undertaken; instead, we interpret the HDL-cholesterol results as a tentative signal that requires confirmation in future RCTs. Mechanistically, in addition to reducing overall energy intake, the metabolic effects of TRE in PMOS may partially relate to improved alignment between eating–fasting cycles and endogenous circadian rhythms, but these pathways remain incompletely delineated [11,13,23].

### Strengths, Limitations, and Future Directions

The current study has several clinically relevant strengths. Across the included studies, TRE was generally well tolerated and associated with high adherence across the included studies, suggesting that it may represent a feasible dietary approach for women with PMOS. However, whether TRE offers superior long-term adherence or sustainability compared with conventional CR remains uncertain. For many women with PMOS—particularly those balancing academic, occupational, and family responsibilities—restricting food intake to defined eating windows may impose a lower psychological and behavioral burden than continuous calorie monitoring. Beyond its potential metabolic benefits, TRE may therefore offer important adherence-related advantages that support its long-term applicability in PMOS management.

Taken together, these findings should be interpreted as preliminary signals rather than definitive evidence. The overall certainty of evidence is limited by the very small number of RCTs, modest sample sizes, short follow-up durations, heterogeneity in TRE protocols and comparators, and some risk of bias concerns. As a result, the observed improvements in insulin-sensitivity indices and selected lipid and hormonal measures should be viewed as hypothesis-generating, and further confirmation in larger, longer-term, and methodologically rigorous trials is required before firm clinical inferences can be made. The available evidence is constrained by the relatively small number of RCTs and participants, which may reduce the statistical power to detect modest between-group differences. In addition, the limited number of studies precluded meaningful assessment of publication bias and restricts the ability to conduct subgroup or meta-regression analyses. Nevertheless, it is plausible that between-study differences in TRE implementation (eating-window duration and clock time, with or without prescribed energy restriction), intervention duration, baseline adiposity and insulin resistance, and concomitant pharmacological treatments contributed to the heterogeneity observed in anthropometric and hormonal outcomes. Dietary intake and intervention adherence were assessed primarily through self-reported food records and adherence logs, rendering the included studies susceptible to recall bias and underreporting of energy intake. Future studies should incorporate more objective methods for monitoring dietary adherence and eating behaviors to improve the accuracy and reliability of behavioral assessments. In addition, whether TRE improves long-term adherence or sustainability compared with conventional calorie restriction remains uncertain and warrants evaluation in longer-duration trials incorporating objective measures of adherence.

Another important consideration is the potential of behavioral overlap within control groups. As highlighted by Corapi et al., participants assigned to control conditions may unintentionally modify their eating windows during the study period, thereby attenuating the observed intervention effects [25]. Future protocols should therefore clearly define and actively monitor control eating windows (e.g., ≥12 h) to better isolate the effects of fasting. Recruitment also posed substantial challenges across trials, likely reflecting the demanding academic, occupational, and family responsibilities commonly experienced by young women with PMOS. To enhance feasibility and long-term adherence, future investigations may benefit from incorporating decentralized visits, remote monitoring, telehealth-based follow-up, flexible scheduling, and financial incentives, as proposed in the TimeMAP trial [26]. Additionally, future RCTs should incorporate direct measures of circadian function, such as melatonin secretion patterns, sleep timing, or chronotype assessments, to better elucidate the mechanisms underlying the metabolic and hormonal effects of TRE in women with PMOS. Finally, important outcomes relevant to PMOS pathophysiology were insufficiently evaluated across the included studies. Data on body composition, particularly fat distribution and visceral adiposity, were largely unavailable despite their central role in the metabolic dysfunction of PMOS. Likewise, assessment of the broader reproductive endocrine profile, including LH and FSH, was limited. Future trials should incorporate comprehensive evaluations of both body composition and reproductive hormonal outcomes to better characterize the effects of TRE in this population.

## 5. Conclusions

PMOS is a complex metabolic disorder affecting a substantial proportion of women worldwide. Our findings suggest that TRE may reduce insulin resistance and increase HDL cholesterol levels in this population, while effects on other metabolic, anthropometric, and hormonal outcomes were generally neutral. Given the small number of short-term trials and modest sample sizes, these findings should be regarded as preliminary, and the overall certainty of evidence is limited. TRE also showed high adherence across trials, suggesting that it may be a feasible dietary approach; however, its long-term acceptability and sustainability relative to conventional calorie restriction remain to be established. Overall, current evidence provides a promising signal that TRE may benefit women with PMOS, but larger and longer-term RCTs are required before firm clinical conclusions or practice recommendations can be made.

## Figures and Tables

**Table 1:** Characteristics of randomized controlled trials of time-restricted eating in women with PMOS.

Study	Country	Sample Size (N) Groups (n)	Study Design	Age	Baseline BMI (kg/m^2^)	TRE Protocol	Control Diet	Study Duration
Corapi et al., 2026 [25]	USA	N = 52 TRE (n = 26) CR (n = 26)	Parallel	TRE: 30 ± 6 CR: 28 ± 4	TRE: 35 ± 7 CR: 35 ± 7	TRE: 18:6 (6 h eating window, 13:00–19:00) Ad libitum intake	Daily CR (25% energy restriction)	6 months
Talebi et al., 2024 [24]	Iran	N = 60 TRE (n = 30) CR (n = 30)	Parallel	TRE: 30 ± 5 DCR: 31 ± 5	TRE: 29.6 ± 3.4 DCR: 30.6 ± 3.5	TRE: 14:10 (10 h eating window, 8:00–18:00) Ad libitum intake	Daily CR (25% energy restriction)	2 months
Zheng et al., 2025 [23]	China	N = 93 TRE (n = 46) CR (n = 47)	Parallel	TRE: 26.1 ± 4.8 Control: 27.2 ± 5.6	TRE: 30.7 ± 3.9 Control: 30.2 ± 4.1	TRE: 16:8 (8 h eating window, 8:00–16:00 or 9:00–17:00) CR1200–1500 kcal/day	CR 1200–1500 kcal/day (47)	4 months
Floyd et al., 2026 [26]	Ireland	N = 11	Crossover	27 (23–30)	35 (30.4–41.35)	TRE: 18:6 (6 h eating window, 8:00–14:00 or 12:00–18:00, or any time between) Ad libitum intake	Ad libitum	3 months

**Footnotes:** TRE: time-restricted eating; CR: calorie-restriction; BMI, body mass index; TRE ratios (e.g., 16:8) denote fasting: eating hours per 24 h period. Age and BMI are provided as mean ± standard deviation for all studies, except Floyd et al., provided these data as median (interquartile range).

## Data Availability

No new data were created or analyzed in this study, Data Sharing is not applicable to this article.

## Supplementary Materials

The following supporting information can be downloaded at: https://www.mdpi.com/article/10.3390/nu18132096/s1, Table S1: Database Search strategy; Table S2: Excluded studies with reason; Figure S1: Risk of bias summary; Figure S2: Forest plots for the hirsutism of time-restricted eating (TRE) vs. control.
