# Supplementary material for: The Effects of Time-Restricted Eating in Women with Polyendocrine Metabolic Ovarian Syndrome: A Systematic Review and Meta-Analysis
Source: Nutrients. 2026 Jun 26;18(13):2096. doi: 10.3390/nu18132096 (PMC13363535; doi:10.3390/nu18132096)

Supplementary Table S1: Database Search strategy

	Keywords	Results
PubMed	("polycystic ovary syndrome"[Title/Abstract] OR PCOS[Title/Abstract] OR "polycystic ovary"[Title/Abstract] OR "polycystic ovarian syndrome"[Title/Abstract]) AND ("time-restricted eating"[Title/Abstract] OR "time restricted eating"[Title/Abstract] OR "time-restricted feeding"[Title/Abstract] OR "time restricted feeding"[Title/Abstract] OR TRE[Title/Abstract] OR TRF[Title/Abstract] OR "16:8"[Title/Abstract] OR "16 8"[Title/Abstract])	25
Scopus	TITLE-ABS-KEY ("polycystic ovary syndrome" OR PCOS OR "polycystic ovary" OR "polycystic ovarian syndrome") AND ("time-restricted eating" OR "time restricted eating" OR "time-restricted feeding" OR "time restricted feeding" OR TRE OR TRF OR "16:8" OR "16 8")	73
Web of Science	("polycystic ovary syndrome" OR PCOS OR "polycystic ovary" OR "polycystic ovarian syndrome") AND ("time-restricted eating" OR "time restricted eating" OR "time-restricted feeding" OR "time restricted feeding" OR TRE OR TRF OR "16:8" OR "16 8")	113
Embase	('polycystic ovary syndrome':ab OR 'polycystic ovarian syndrome':ab OR pcos:ab OR 'polycystic ovary disease':ab OR 'polycystic ovarian disease':ab) AND ('intermittent fasting':ab OR 'time restricted eating':ab OR 'time restricted feeding':ab OR tre:ab OR trf:ab OR '16:8':ab OR '14:10':ab OR '18:6':ab OR '20:4':ab) AND ('clinical trial':ab OR 'randomized controlled trial':ab OR rct:ab)	28

Supplementary Table S2: Excluded studies with reason

Study title	Reason for exclusion
Intermittent Fasting Diet Versus Resistive Exercise Program on Insulin Resistance in Obese Women With P.C.O.S	Trial registration
Study of Time-restricted Eating (TRE) to Clinical Pregnancy Rate Via IVF/ICSI in Women With PCOS and Glucose Metabolism Disorder: A Multicenter Randomized Controlled Trial (RCT)	Trial registration
Time-Restricted Eating for the Management of Polycystic Ovary Syndrome-A Randomized Controlled Trial	Abstract
TIME RESTRICTED EATING FOR THE MANAGE-MENT OF POLYCYSTIC OVARY SYNDROME: A RAN-DOMIZED CONTROLLED TRIAL	Abstract
Time Restricted Eating for Polycystic Ovary Syndrome Management: A Randomized Controlled Trial	Abstract
The Effect of Time-restricted Eating on Insulin Levels in Polycystic Ovarian Syndrome: A Randomised Feasibility Study of Real-world Clinical Advice	Abstract
Intermittent Fasting as a Nonpharmacologic Strategy to Manage Polycystic Ovary Syndrome	Not RCT
Eight-Hour Time-Restricted Feeding: A Strong Candidate Diet Protocol for First-Line Therapy in Polycystic Ovary Syndrome.	Not RCT
Eight-hour time-restricted feeding improves endocrine and metabolic profiles in women with anovulatory polycystic ovary syndrome.	Not RCT
Comparison of the impact of intermittent fasting diet alone or in conjunction with probiotic supplementation versus calorie-restricted diet on inflammatory, oxidative stress, and antioxidant capacity biomarkers in women with polycystic ovary syndrome: A r	Duplicate
Beyond Hormones: The Efficacy of Time-Restricted Eating as an Alternative to Traditional Pharmacological Therapy for Polycystic Ovary Syndrome	Not peer-reviewed

Supplementary Figure S1: Risk of bias summary

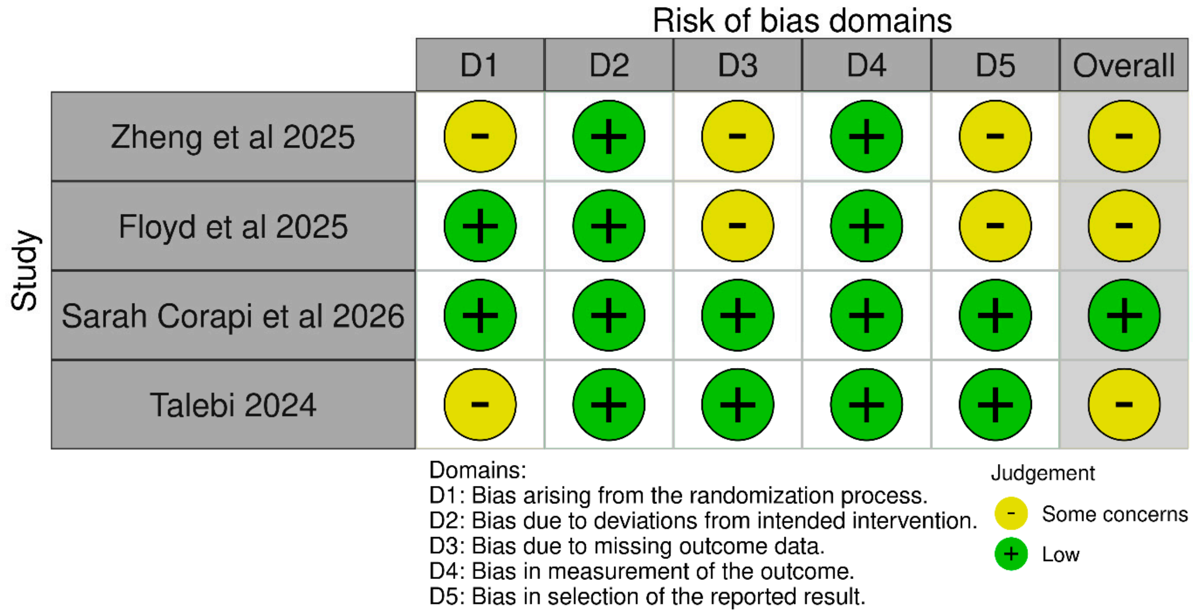

Supplementary Figure S2: Forest plots for the hirsutism of time-restricted eating (TRE) vs. control.

	Experimental			Control				Std. Mean Difference
Study or Subgroup	Mean	SD	Total	Mean	SD	Total	Weight	IV, Fixed, 95% CI
Corapi et al., 2026	-1.75	7	26	-0.75	6.17	26	25.5%	-0.15 [-0.69, 0.40]
Talebi et al., 2024	-3	3	30	-2	2	30	28.9%	-0.39 [-0.90, 0.12]
Zheng et al., 2025	-0.53	1.45	46	-0.43	1.45	47	45.6%	-0.07 [-0.48, 0.34]
**Total (95% CI)**			**102**			**103**	**100.0%**	**-0.18 [-0.46, 0.09]**

Heterogeneity: Chi² = 0.93, df = 2 (P = 0.63); I² = 0%
Test for overall effect: Z = 1.29 (P = 0.20)

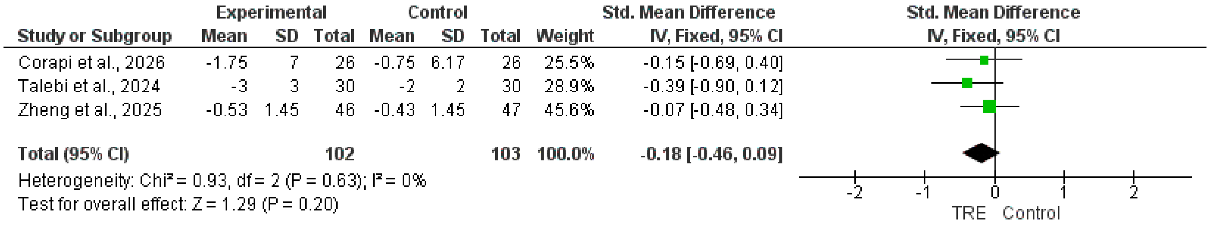

Supplement: Supplementary file 1 [file nutrients-18-02096-s001.zip › nutrients-4364568-supplementary.pdf]
